# Structural and biochemical insights into the catalytic mechanisms of two insect chitin deacetylases of the carbohydrate esterase 4 family

**DOI:** 10.1074/jbc.RA119.007597

**Published:** 2019-02-12

**Authors:** Lin Liu, Yong Zhou, Mingbo Qu, Yu Qiu, Xingming Guo, Yuebin Zhang, Tian Liu, Jun Yang, Qing Yang

**Affiliations:** From the ‡ State Key Laboratory of Fine Chemical Engineering, School of Life Science and Biotechnology and School of Software, Dalian University of Technology, Dalian 116024, China,; § Department of Protein Engineering, Biologics Research, Sanofi, Bridgewater, New Jersey 08807,; the ¶ Laboratory of Molecular Modeling and Design, State Key Laboratory of Molecular Reaction Dynamics, Dalian Institute of Chemical Physics, Chinese Academy of Sciences, Dalian 116024, China, and; the ‖ State Key Laboratory for Biology of Plant Diseases and Insect Pests, Institute of Plant Protection, Chinese Academy of Agricultural Sciences, Beijing 100193, China

**Keywords:** chitin, carbohydrate, carbohydrate-binding protein, protein structure, Bombyx mori, structural biology, carbohydrate esterase, chitin deacetylase, chitosan, deacetylation activity, SeMet single-wavelength anomalous diffraction

## Abstract

Insect chitin deacetylases (CDAs) catalyze the removal of acetyl groups from chitin and modify this polymer during its synthesis and reorganization. CDAs are essential for insect survival and therefore represent promising targets for insecticide development. However, the structural and biochemical characteristics of insect CDAs have remained elusive. Here, we report the crystal structures of two insect CDAs from the silk moth *Bombyx mori*: *Bm*CDA1, which may function in cuticle modification, and *Bm*CDA8, which may act in modifying peritrophic membranes in the midgut. Both enzymes belong to the carbohydrate esterase 4 (CE4) family. Comparing their overall structures at 1.98–2.4 Å resolution with those from well-studied microbial CDAs, we found that two unique loop regions in *Bm*CDA1 and *Bm*CDA8 contribute to the distinct architecture of their substrate-binding clefts. These comparisons revealed that both *Bm*CDA1 and *Bm*CDA8 possess a much longer and wider substrate-binding cleft with a very open active site in the center than the microbial CDAs, including *Vc*CDA from *Vibrio cholerae* and *Ar*CE4A from *Arthrobacter* species AW19M34-1. Biochemical analyses indicated that *Bm*CDA8 is an active enzyme that requires its substrates to occupy subsites 0, +1, and +2 for catalysis. In contrast, *Bm*CDA1 also required accessory proteins for catalysis. To the best of our knowledge, our work is the first to unveil the structural and biochemical features of insect proteins belonging to the CE4 family.

## Introduction

Carbohydrate esterase 4 family (CE4)^2^ chitin deacetylases (CDAs, EC 3.5.1.41) catalyze the removal of acetyl groups from chitin to form chitosan (1), a polymer of β-(1,4)-linked d-glucosamine residues. CDAs are widely distributed in protists, diatoms, bacteria, fungi, nematodes, and insects (2–8), playing vital roles in chitinous matrix formation and modification (9), as well as in biological attack of fungal pathogens (5, 10). CDAs have been considered promising targets for the design of antifungal, antibacterial, and pest control reagents (2, 11–13).

To date, six crystal structures of CDAs from fungi and bacteria have been determined, including *Cl*CDA from *Colletotrichum lindemuthianum* (14), *An*CDA from *Aspergillus nidulans* (15), *Ar*CE4A from *Arthrobacter* species AW19M34-1 (16), two chitooligosaccharide deacetylases *Vc*CDA from *Vibrio cholerae* (17), *Vp*CDA from *Vibrio parahemeolyticus* (18), and one putative CDA (*Ec*CDA) from *Encephalitozoon cuniculi* (19). Except the structure of *Ec*CDA, which is not capable of deacetylating chitin, the other structures exhibit conserved critical residues in the active site, indicating that all CDAs use the same metal-assisted general acid/base catalytic mechanism conserved across CE4 enzymes (14–18, 20–28). However, the large discrepancies in the shape of the substrate-binding site among these enzymes lead to varied substrate preferences and deacetylation modes. The crystal structures of *Vc*CDA and *Ar*CE4A represent the only two known CDA structures resolved in the presence of their oligosaccharide substrates (16, 17). The *Vc*CDA–substrate complex structures provide evidence that six critical loops adopt conformational changes to effectively trap chitooligosaccharides in the substrate-binding pocket. *Vp*CDA has a substrate-binding pocket nearly identical to that of *Vc*CDA and is highly active toward (GlcNAc)_2_ (18, 29). In contrast, there are marked differences in the loops surrounding the substrate-binding site in *Ar*CE4A, *Cl*CDA, and *An*CDA (16, 17). All of them adopt shorter loops to form a relatively open substrate-binding cleft and are active toward both chitooligosaccharide and chito-polysaccharide substrates. Sequence alignment indicates that insect CDAs do not share the specific loops observed in other CDAs (30, 31). Unfortunately, the structures of insect CDAs have long been pursued without success.

Insects possess a greater number of CDAs than any other organism. An insect may possess as many as five groups of genes encoding CDAs (32), which may function in the epidermis, tracheal tubes, and midgut (33–36). Previous research suggested that chitosan was present in the precise locations where the flexibility of chitin fibers was required (37). Two *cda* gene mutants of *Drosophila melanogaster* embryos resulted in elongated and tortuous tracheal tubes (34, 35). RNAi of all nine *cda* genes from *Tribolium castaneum* resulted in abnormal phenotypes in the tracheal tubes and cuticle and in joint defects, followed by molting failure or even death (33). RNAi of *LmCDA2* from *Locusta migratoria* changed the chitin organization in the procuticle from a helicoidal to a unidirectional orientation (38). However, many efforts have failed to demonstrate the deacetylation activities of insect CDAs toward chitinous substrates *in vitro* (30, 39). Data about the biochemical characteristics and structure–function relationship of insect CDAs remain scarce.

In this study, two CDAs from *Bombyx mori*, *Bm*CDA1 and *Bm*CDA8, were crystallized and resolved, providing the first insight into the structural characteristics, activity profiles, and deacetylation mode of insect CDAs. The structures revealed that insect CDAs possess several unique structural features that distinguish them from other known CE4 enzymes. This work will also assist the development of specific agrochemicals for pest control.

## Results

### Overall structure of BmCDA1-CAD

**Figure 1.**
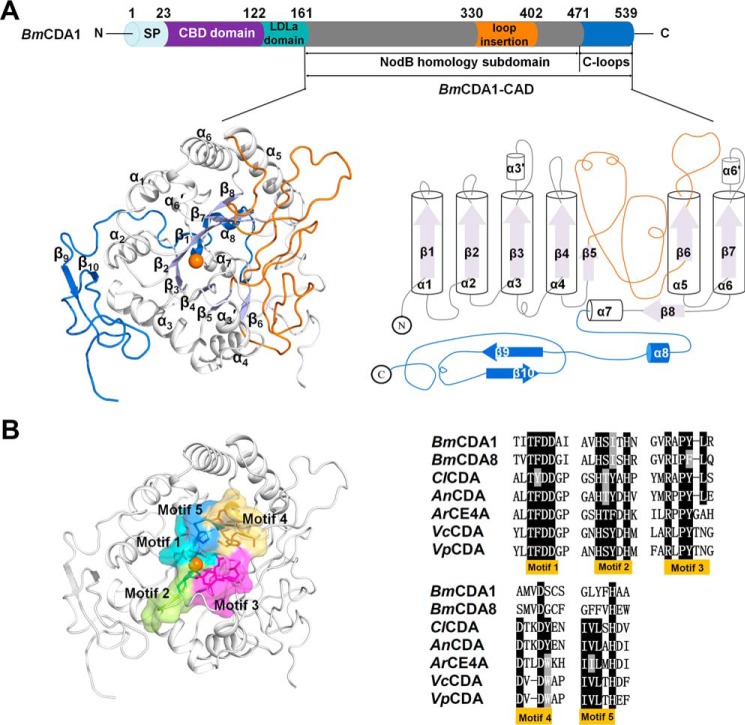
**Domain architecture, overall structure, and active site of *Bm*CDA1.** *A*, color-coded domain organization of full-length *Bm*CDA1 (*upper panel*) and cartoon representations of the architecture of the NodB homology subdomain (*slate*, barrel; *white*, α-helices), C-terminal loops (*blue*), and loop insertion (*orange*) of *Bm*CDA1-CAD (*lower panel*). *SP*, signal peptide. The zinc iron is represented as an *orange sphere. B*, surface representations of the active site (*left panel*). Motifs 1–5 of the active site are colored *cyan* (motif 1), *lime green* (motif 2), *magenta* (motif 3), *yellow* (motif 4), and *blue* (motif 5). The key residues in the five motifs are shown in stick representation using the same color scheme. Structure-based sequence alignment of five CDAs showing the conserved motifs 1–5 (*right panel*). The highly conserved residues are indicated.

*Bm*CDA1 is composed of an N-terminal signal region (residues 1–23), a chitin-binding domain (residues 24–122), a low-density lipoprotein receptor domain (residues 123–161), and a catalytic domain (CAD, residues 162–539) (Fig. 1*A*). Our experiments showed that full-length recombinant *Bm*CDA1 was not stable and underwent autocleavage when incubated with the crystallization reagent. Thus, the truncated form of *Bm*CDA1 (*Bm*CDA1-CAD, residues 162–539) was cloned, expressed, and purified for crystallization. Diffraction data were collected to 1.98 and 2.4 Å on native and SeMet protein crystals, respectively, and the structure was solved using the SeMet single-wavelength anomalous diffraction technique.

*Bm*CDA1-CAD was crystallized in the trigonal space group *I4* with one molecule in the asymmetric unit (Table 1). The overall structure of *Bm*CDA1-CAD consists of two regions: a CE4-conserved NodB homology subdomain and C-terminal loops (Fig. 1*A*). The NodB homology domain is a (β/α)_7_ barrel (residues 162–471) composed of seven parallel β-strands arranged in a barrel that is surrounded by six α-helices. Notably, the (β/α)_7_ barrel contains one loop insertion (residues 331–402) between β_5_ and α_5_. The C-terminal loops (residues 472–539) consist of α_8_, a pair of antiparallel β-strands (β_9_ and β_10_), and several loops. These structural elements, the loop insertion of the (β/α)_7_ barrel and the C-terminal loops, appear to be unique because they are not present in any of the other CE4 structures determined to date.

**Table 1:** **Data collection and structural refinement statistics** The values in parentheses are for highest-resolution shell.

	Se-*Bm*CDA1-CAD	Native-*Bm*CDA1-CAD	*Bm*CDA8
**Data collection**			
Wavelength (Å)	0.979452	0.97776	0.97775
Resolution (Å)	2.33–50 (2.33–2.41)	1.98–50 (1.98–2.01)	2.30–50 (2.30–2.34)
Temperature (K)	100	100	100
Space group	I4	I4	P3_2_21
*a*, *b*, *c* (Å)	134.941, 134.941, 77.120	136.006, 136.006, 77.209	115.017, 115.017, 106.510
α, β, γ (°)	90, 90, 90	90, 90, 90	90, 90, 120
Unique reflections	29,817 (2917)	49,155 (2407)	36,623 (1815)
Completeness (%)	99.9 (98.8)	100 (100)	100 (100)
*R*_sym_ (%)	7.7 (20)	15.2 (83.5)	15.4 (75.1)
Redundancy	15.0 (14.6)	13.5 (12.7)	11.2 (10.5)
*I*/σ(*I*)	15.9 (14.2)	4.3 (2.5)	3.0 (2.1)
CC_½_	0.991	0.826	0.834
Wilson B factor (Å^2^)	26.86	23.59	37.59

**Statistics for refinement**			
Resolution (Å)	2.396–42.67 (2.396–2.482)	1.98–47.78 (1.98–2.05)	2.399–39.07 (2.399–2.485)
No. of reflections	27317 (2673)	49077 (4834)	28971 (2523)
Completeness (%)	99.8 (99.14)	99.81 (99.42)	89.7 (79.67)
*R*_work_/*R*_free_ (%)	16.27 (18.17)/19.09 (23.56)	16.66 (23.89/18.96 (26.97)	16.97 (21.18)/18.88 (23.79)
Average *B* factor (Å^2^)	36.2	30.55	43.67
Protein atoms	3095 (35.64)	3075 (29.33)	2907 (43.31)
Ligand	43 (54.45)	43 (50.66)	29 (62.94)
Water molecules	236 (40.22)	399 (37.84)	170 (46.43)
Other atoms	0	0	0
RMSD			
Bond angles (°)	1.03	0.99	1.02
Bond length (Å)	0.01	0.01	0.013
Ramachandran plot (%)			
Favored region	96.3	96.8	97.8
Allowed region	3.7	3.2	2.2
Outliers	0	0	0
Protein Data Bank code	5ZNS	5ZNT	5Z34

### Active site and substrate-binding cleft of BmCDA1-CAD

**Figure 2.**
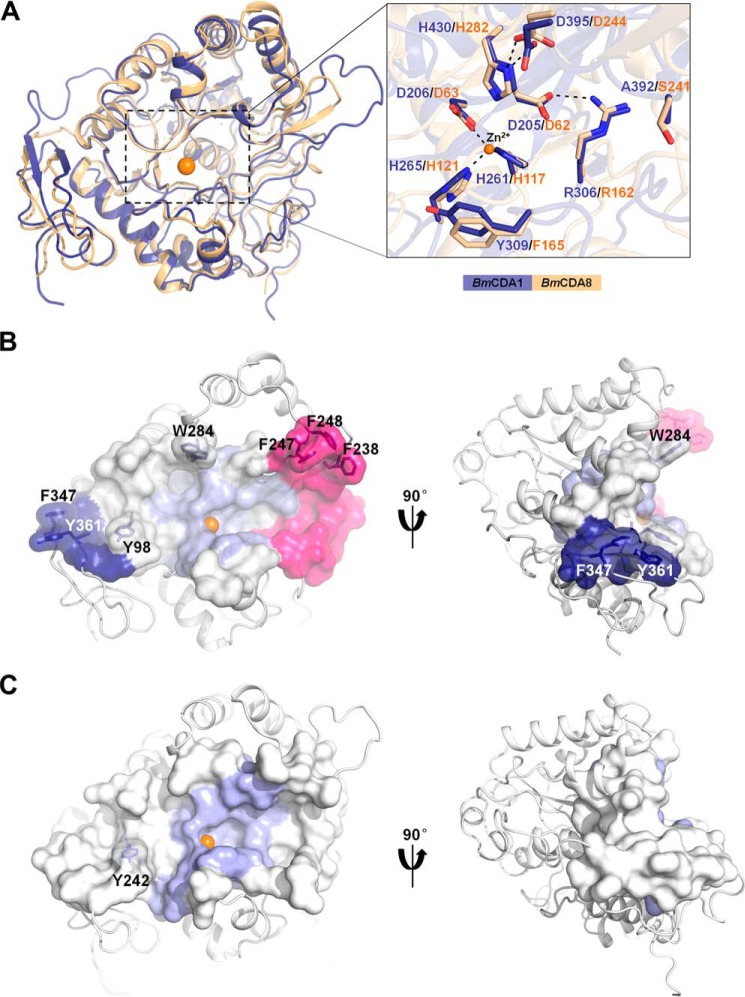
**Structural comparison of *Bm*CDA1 and *Bm*CDA8.** *A*, structural alignment of the overall structure (*left panel*) and active site (*right panel*) of *Bm*CDA1 and *Bm*CDA8. *B* and *C*, substrate-binding clefts of *Bm*CDA8 (*B*) and *Bm*CDA1 (*C*). The substrate-binding clefts are shown in surface representation, whereas the remaining regions are shown in cartoon representation. In *Bm*CDA8, the surfaces are colored *slate* (active site), *deep blue* (residues from the C-terminal loops), *hot pink* (residues from the loop insertion), and *white* (other regions). The surface-exposed aromatic residues that line in *Bm*CDA8 and *Bm*CDA1 are shown in stick representation in *black*. The structures of the substrate-binding clefts are each viewed from two angles rotated 90° along the vertical axes.

The active site of *Bm*CDA1-CAD is located at the top center of the (β/α)_7_ barrel and contains a metal-binding triad conserved across the CE4 family, namely, a zinc ion coordinated by Asp^206^, His^261^, and His^265^ (Figs. 1*B* and 2*A*). Like most CE4 family members, *Bm*CDA1-CAD contains an active site shaped by five motifs, motifs 1–5 (Fig. 1*B*). Motif 1 (TFDD) contains the catalytic base Asp^205^ and the zinc-binding residue Asp^206^. Motif 2 (HSITH) contains two zinc-binding residues, His^261^ and His^265^. Motif 3 (RAPYL) contains the canonical Arg^306^ responsible for stabilizing the catalytic base Asp^205^. Structure-based sequence alignment indicates that motif 4 (AMVDS) is less conserved because most CE4 enzymes possess a motif 4 with the sequence D*XX*D(W/Y) (Fig. 1*B*). As the residue Trp/Tyr forms one wall of the active pocket, the replacement of Trp/Tyr by Ser results in an open active site for *Bm*CDA1. Motif 5 (YFH) is also less conserved because motif 5 of most CDAs contains L*X*H. The canonical Leu contributes to form a hydrophobic patch (21). Taken together, the differences in motif 4 and motif 5 confer *Bm*CDA1 a more open and wider active pocket.

A long substrate-binding cleft was observed on the surface of *Bm*CDA1-CAD (Fig. 2*C*), with only one solvent-exposed hydrophobic residue (Tyr^242^) that might aid in substrate binding. The substrate-binding cleft is shallow and open when compared with the other microbial CDAs because of the lack of several loops that give microbial CDAs their characteristically deep and narrow substrate-binding cleft (Fig. S1).

### Crystal structure of BmCDA8

*Bm*CDA8 (residues 19–381) lacking the N-terminal signal peptide was expressed, purified, and crystallized. Diffraction data were collected to 2.4 Å. The structure was resolved by molecular replacement with *Bm*CDA1-CAD as the search model. *Bm*CDA8 was crystallized in the trigonal space group *P3*_2_*21* with one molecule in the asymmetric unit (Table 1). Residues 19–22 were not included in the final structure because of a lack of interpretable electron density. *Bm*CDA8 showed 37% sequence identity with *Bm*CDA1-CAD. The overall architecture of *Bm*CDA8 was similar to that of *Bm*CDA1-CAD (Fig. 2*A*), corresponding to an root-mean-square deviation (RMSD) of 1.31 Å for 345 equivalent C^α^ atoms.

Similar to *Bm*CDA1, *Bm*CDA8 (residues 23–381) consists of two regions: a CE4-conserved NodB homology subdomain and C-terminal loops. The active site of *Bm*CDA8 is located at the top center of the (β/α)_7_ barrel (Fig. 2*A*). The conserved metal-binding triad is Asp^63^–His^117^–His^121^ coordinated with a zinc ion, and the active site is shaped by motifs 1–5 (Figs. 1*B* and 2*A*).

However, two differences between *Bm*CDA8 and *Bm*CDA1-CAD were observed. One obvious difference is the replacement of Ala^392^ (*Bm*CDA1) by Ser^241^ (*Bm*CDA8) in motif 4. The other difference is the substrate-binding cleft. Unlike *Bm*CDA1-CAD, *Bm*CDA8 contains a narrower and deeper substrate-binding cleft that passes through the active site where the catalytic reaction occurs (Fig. 2, *B* and *C*). As shown in Fig. 2*B*, this cleft has an extended structure with two open ends. One end is shaped by the loop insertion. In detail, surface-exposed Phe^238^, Phe^247^, and Phe^248^ form a hydrophobic “claw” at the top. The other end is shaped by the C-terminal domain that provides two aromatic residues, Phe^347^ and Tyr^361^.

### Enzymatic activity and the deacetylation mode of BmCDA8

The activity assay indicated that *Bm*CDA8 instead of *Bm*CDA1 is active. To investigate the catalytic characteristics of *Bm*CDA8, the enzymatic activity was determined using various kinds of chitinous substrates, including (GlcNAc)_1–6_, and the polymeric substrates ethylene glycol chitin and colloidal chitin. Among oligomeric chitinous substrates, *Bm*CDA8 showed no activity toward (GlcNAc)_1–2_ but did exhibit activities toward (GlcNAc)_3–6_. As for (GlcNAc)_3–6_, the *k*_cat_/*K_m_* values of *Bm*CDA8 increased as the degree of polymerization increased. In contrast to its relatively low affinities toward chitooligosaccharides, *Bm*CDA8 showed higher affinities toward the polymeric substrates ethylene glycol chitin and colloidal chitin (Table 2).

**Table 2:** **Kinetic parameters of *Bm*CDA8**

Substrate	*K_m_*		*V*_max_	*k*_cat_	*k*_cat_/*K_m_*
	*mm*	*mg ml*^−*1*^	*mm min*^−*1*^	*min*^−*1*^	*min*^−*1*^ *mm*^−*1*^
GlcNAc					
(GlcNAc)_2_					
(GlcNAc)_3_	76.7	48.1	0.0162	7.62	0.099
(GlcNAc)_4_	12.3	10.2	0.0119	5.59	0.45
(GlcNAc)_5_	15.7	16.2	0.0160	7.52	0.48
(GlcNAc)_6_	9.2	11.4	0.0193	9.07	0.98

	*K_m_*	*V*_max_	*k*_cat_	*k*_cat_/*K_m_*
	*mg ml*^−*1*^	*mg ml min*^−*1*^	*min*^−*1*^	*min*^−*1*^ *ml mg*^−*1*^
EGC*^a^*	1.926	0.085	0.097	0.050
Colloidal chitin	1.599	0.011	0.012	0.0077

*^a^* EGC, ethylene glycol chitin.

**Figure 3.**
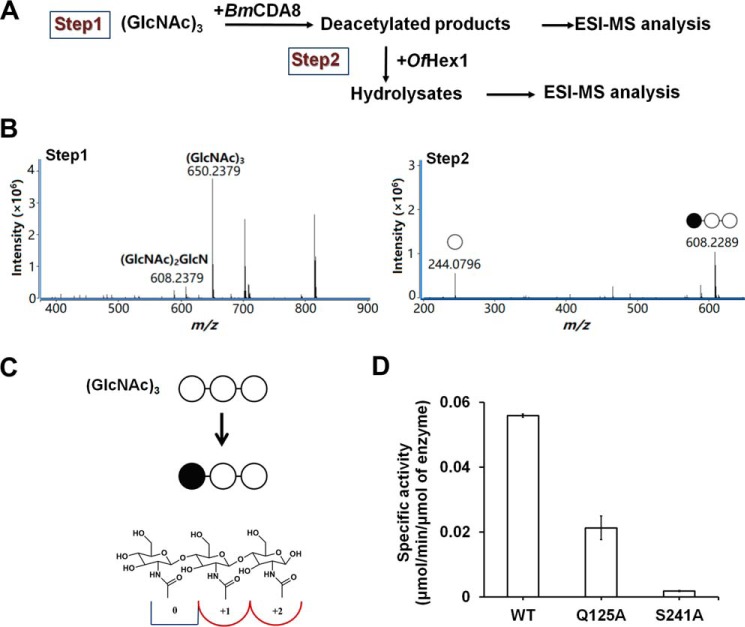
**Structural analysis of deacetylated products of (GlcNAc)_3_.** *A*, scheme of the two-step analysis method. *B*, ESI-TOF MS spectra of the deacetylated products of (GlcNAc)_3_. *C*, schematic representations of the binding mode of (GlcNAc)_3_ to *Bm*CDA8. ○, GlcNAc residue; ●, GlcN residue. *D*, specific activities of the WT protein and mutants of *Bm*CDA8 toward (GlcNAc)_3_.

The deacetylation mode of *Bm*CDA8 was investigated by a two-step analysis of the deacetylated products of (GlcNAc)_3_ (Fig. 3*A*). In the first step, electrospray ionization (ESI)–MS analysis was performed to determine the number of deacetylated GlcNAc residues. In the second step, ESI-MS analysis was performed again to determine the deacetylation sites. Before the ESI-MS analysis, the deacetylated products were pretreated with the enzyme *Ostrinia furnacalis* hexosaminidase1 (*Of*Hex1), which specifically cleaves β-1,4–linked GlcNAcs instead of GlcNs from the nonreducing ends of the chitooligosaccharides. Thus, only chitooligosaccharides with a GlcN at the nonreducing end remained for the second ESI-MS analysis. For the substrate (GlcNAc)_3_, the first-step ESI-MS analysis showed that the products included (GlcNAc)_3_ and a monodeacetylated product with a mass loss of 42.0, the mass of a CH_3_CO-H group. The monodeacetylated product was not further hydrolyzed by *Of*Hex1 in the second step, indicating that the product was GlcN—GlcNAc–GlcNAc (Fig. 3*B*). Notably, neither GlcN nor monodeacetylated products of (GlcNAc)_2_ were present, indicating that deacetylation occurred at the first GlcNAc at the nonreducing end. Because the biochemical data indicated that *Bm*CDA8 was not able to deacetylate GlcNAc or (GlcNAc)_2_ (Table 2), we deduced that *Bm*CDA8 activity requires substrates to occupy subsites 0, +1, and +2 (Fig. 3*C*), where 0 is the catalytic site, and the plus sign refers to the reducing ends, according to the nomenclature commonly used for CE4 enzymes (40–42).

To further confirm the requirement of the subsites for catalysis, mutagenesis of Gln^125^ at +1 subsite and Ser^241^ at +2 subsite was performed. The two substrate-binding sites were predicted by molecular dynamics stimulations (supporting information, Fig. S4). The activity of the mutants toward (GlcNAc)_3_ was determined. As shown in Fig. 3*D*, both Q125A and S241A showed markedly lower specific activity than the WT protein. The mutation of Ser^241^ caused much more serious impairment of activity than the mutation of Gln^125^, even though Gln^125^ interacts with both subsites +1 and +2. This difference suggests that subsite +2 might be more crucial for (GlcNAc)_3_ binding.

### Enzymatic activity of BmCDA1 requires accessary proteins

**Figure 4.**
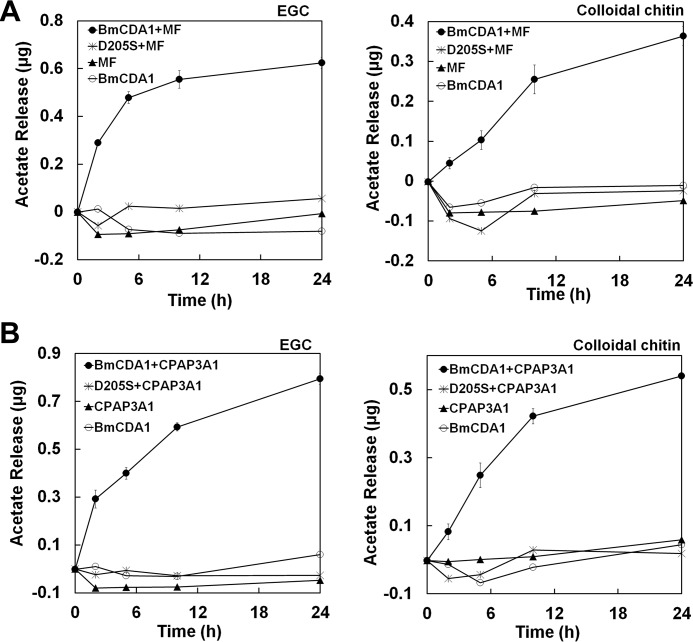
**The requirement of accessary proteins for *Bm*CDA1 to achieve activity.** The addition of MF (*A*) and CPAP3-A1 (*B*) substantially stimulated *Bm*CDA1 activity toward ethylene glycol chitin (*EGC*, *left panel*) and colloidal chitin (*right panel*).

Our study showed that the deacetylation activity of *Bm*CDA1 was undetectable toward various chitinous substrates even with prolonged incubation time to 120 h or with higher enzyme concentrations at 200 μm. To understand the activation mechanism of *Bm*CDA1, we added molting fluid (MF), which is a protein mixture secreted by insect epidermal cells that facilitates old cuticle shedding, into the reaction mixture. Strikingly, the activity of *Bm*CDA1 toward ethylene glycol chitin and colloidal chitin was boosted in the presence of MF when compared with that of *Bm*CDA1 alone and the catalytic residue-mutated form D205S (Fig. 4*A*). To further understand what proteins are involved in the activation of *Bm*CDA1, the cuticular chitin-binding protein CPAP-3A1, which is a homolog of obstructor A in *Drosophila* that physically interacts with Serpentine (the CDA1 homolog in *Drosophila*) (43), was mixed with an equimolar amount of *Bm*CDA1. The enzymatic activity assay showed a significant increase in the deacetylation activity of *Bm*CDA1 in the presence of CPAP3-A1 (Fig. 4*B*). The *in vitro* pulldown assay illustrated that CPAP3-A1 can pull down *Bm*CDA1 (Fig. S2). However, CPAP3-D, which belongs to the same CPAP family as CPAP3-A1 and shows the highest binding affinity to deacetylated chitin among CPAP family members (44), could not activate nor pull down *Bm*CDA1 (data not shown). Taken together, these data indicated that *Bm*CDA1 requires specific accessary proteins to achieve activity.

## Discussion

This study on *Bm*CDA1 and *Bm*CDA8 provides the first structural and biochemical comparisons of insect CDAs. As revealed by chemical and spectroscopic analyses, chitin in insects is deacetylated at a degree of 5–25% (45). The deacetylation activity of insect CDAs appears to be necessary because abrogation of its activity by gene knockdown results in chitinous laminar organization disorder or even lethality (38, 46). Surprisingly, most studies indicate that insect CDAs are ineffective enzymes in *in vitro* testing assays.

Insect CDAs seem to be designed less active. The deacetylated degree of the insect chitin matrix (5–25% chitosan) was relatively low when compared with that of the fungal cell wall (∼75% chitosan in *Mucor rouxii*) (47). *Mr*CDA from *M. rouxii* was highly active toward chitinous substrates (4). The specific activity of *Mr*CDA toward (GlcNAc)_5_ was 467 μm/min/μm (4), which was 65.7-fold higher than that of *Bm*CDA8. The weak activity of insect CDAs might help to maintain the low degrees of chitin deacetylation observed *in vivo*. Structural alignment of *Bm*CDA1/*Bm*CDA8 with other CE4 enzymes including peptidoglycan deacetylases, poly-β-1,6-*N*-acety-d-glucosamine deacetylases, acetylxylan de-*O*-acetylases, chitin deacetylases, and chitin oligosaccharide deacetylases shows strong conservation of the active site (Fig. S3). The similarity of the active sites suggests that insect CDAs use the same catalytic mechanism. However, the different architectures of the substrate-binding clefts of the CE4 CDAs confer different catalytic properties to these enzymes. The narrow entrance of the substrate-binding pocket of *Vc*CDA is composed of six loops close to each other, contributing a specific and high catalytic efficiency toward chitooligosaccharides (17). The openness and shortness of the substrate-binding cleft of *Ar*CE4A represents a common feature of other CE4 enzymes, conferring them with higher rates for various substrates (16). Unlike *Vc*CDA and *Ar*CE4A, the only two known structures complexed with chitooligosaccharide substrates, both *Bm*CDA1 and *Bm*CDA8 possess a much longer, wider and more open substrate-binding cleft (Fig. 2, *B* and *C*). The lack of steric constraints within the substrate-binding clefts may reduce the effectiveness of trapping substrates once bound, perhaps explaining the weak activity observed for *Bm*CDA1 and *Bm*CDA8. The longer substrate-binding cleft formed by unique loops appears to be an intentional feature of insect CDAs to fit chitin fibers *in vivo*. To illuminate the functions of the unique loops in insect CDAs, we have constructed nine truncates of *Bm*CDA8, each of which lacks partial or whole loops (supporting information, Fig. S5). Unfortunately, we failed to obtain any recombinant proteins. The function of loops unique in insect CDAs requires further investigation.

The activation of *Bm*CDA1 in the presence of molting fluid suggests that the activity of insect CDAs could be regulated by an unknown mechanism. As a component of molting fluid, the chitin-binding protein CPAP3-A1 instead of CPAP3-D1 was capable of activating *Bm*CDA1, suggesting that the activation mechanism is complex. A possibly similar case has been shown for PgaB, a poly-β-1,6-*N*-acety-d-glucosamine deacetylase from *Escherichia coli*. PgaB is composed of two domains: an N-terminal deacetylase domain and a C-terminal GH18/GH20-like domain, the association of which is proposed to create a substrate-binding cleft during de-*N*-acetylation (49). In *Drosophila*, the CPAP3-A1 homolog obstructor A chitin-binding protein interacts with the deacetylation domain protein Serp (CDA1) (43). We further verified the interaction of *Bm*CDA1 and CPAP3-A1 using an *in vitro* pulldown assay. One may deduce the activation of *Bm*CDA1 requires an accessary factor, *e.g.* CPAP3-A1. Future structural studies of the complex will provide information about the activation mechanism.

Taken together, the structural and biochemical data provide insights into the novel characteristics of insect CDAs. The lack of available and clear information regarding insect CE4 enzymes highlights the importance of the addition of the *Bm*CDA1-CAD and *Bm*CDA8 structure to the CE4 structural database, adding new knowledge about the CE4 family.

## Experimental procedures

### Gene cloning and expression plasmid construction

Total RNA was extracted from five *B. mori* specimens at the fifth instar (day 5) using RNAiso^TM^ Plus (TaKaRa, Japan) according to the manufacturer's protocol. The cDNA was synthesized using the PrimeScript^TM^ RT reagent kit (TaKaRa, Japan). The gene encoding *Bm*CDA1-CAD and *Bm*CDA8 was amplified from the cDNA with the primers listed in Table S1. A His_6_ affinity tag was introduced at the C-terminal. The product was ligated into the EcoRI and XhoI restriction sites of the pPIC9 expression vector using the In-Fusion kit (Clontech). The resulting expression plasmids were subsequently linearized with the restriction enzyme SalI to allow integration into the chromosomal DNA of *Pichia pastoris* GS115 (Invitrogen).

### Expression and purification

Recombinant *P. pastoris* was first grown in buffered complex medium containing glycerol (BMGY; Invitrogen) at 301 K to an optical cell density of 4.0 at 600 nm. The cells were collected by centrifugation, resuspended in buffered methanol complex medium (BMMY; Invitrogen), and transferred into a 5-liter fermentation tank. The volume of cultures for production of recombinant proteins is 3 liters. The pH was controlled with a sterilized base solution of 1 m KOH. Protein production was induced by delivering methanol to the vessel at a constant feed rate. The fermentation proceeded for 92 h at 301 K. The culture supernatant was obtained by centrifugation. The supernatant was subjected to ammonium sulfate precipitation with 75% saturation at 277 K for 12 h. After centrifugation, the supernatant was removed, and the precipitate was resuspended in distilled water and then desalted in buffer A (20 mm sodium phosphate, 0.5 m sodium chloride, pH 7.4) using a HiTrap desalting column (5 ml; GE Healthcare). The resulting sample was then loaded into a HisTrap HP affinity column (5 ml; GE Healthcare) equilibrated in buffer A. The target protein was eluted with 20 mm sodium phosphate, 0.5 m NaCl, 250 mm imidazole (pH 7.4). The eluted protein was >95% pure, as analyzed by SDS-PAGE. The purified protein was then desalted in 20 mm Tris (pH 7.4) and 20 mm NaCl and concentrated to an appropriate concentration for the crystallization experiments. The yields for the recombinant proteins are 0.2 mg/liter (*Bm*CDA8), 1 mg/liter (*Bm*CDA1), and 5 mg/liter (*Bm*CDA1-CAD). Mutations were introduced by PCR-based site-directed mutagenesis, and the mutated proteins were purified using the same protocol described above.

### Expression of selenomethionine-containing BmCDA1-CAD

The same strain was used for the expression of SeMet-incorporated *Bm*CDA1-CAD. The growth conditions before induction were the same as those described above. The cells were then washed three times with PBS and resuspended in buffered methanol medium with the following modifications. The medium contained 100 mm potassium phosphate at pH 6.0, 1.34% (w/v) yeast nitrogen base without amino acids (Invitrogen), 0.09 mg/ml adenine sulfate, 0.09 mg/ml uracil, 0.34 mg/ml thiamine, 0.3 mg/ml succinic acid, 0.01 mg/ml inositol, 0.09 mg/ml l-tryptophan, 0.09 mg/ml l-histidine, 0.09 mg/ml l-arginine, 0.09 mg/ml l-tyrosine, 0.09 mg/ml l-leucine, 0.09 mg/ml l-isoleucine, 0.09 mg/ml l-lysine, 0.15 mg/ml l-phenylalanine, 0.3 mg/ml l-glutamic acid, 0.3 mg/ml l-aspartic acid, 0.45 mg/ml l-valine, 0.6 mg/ml l-threonine, 1.2 mg/ml l-serine, 0.12 mg/ml l-cysteine, 0.3 mg/ml l-glutamine, 0.2 mg/ml l-proline, 0.2 mg/ml l-alanine, and 0.1 mg/ml selenomethionine. The induction of the expression and purification procedures were the same as those described above.

### Enzymatic activity assays

The enzyme activity was determined based on the detection of acetate released by the action of *Bm*CDA8. The assays were performed using the K-ACETRM kit (Megazyme International, Wicklow, Ireland) according to the manufacturer's instructions. A deacetylation activity screen was performed using different chitinous substrates, including both polymeric substrates (ethylene glycol chitin (Wako Pure Chemicals), colloidal chitin, and α-chitin (Sigma–Aldrich)) and oligomeric substrates (monomer to hexamer; Qingdao BZ Oligo Biotech Co., Ltd.). The conditions of the enzymatic assay on chitin were as follows: 100 μl of a mixture containing 6 μm enzyme and 0.5 mg of the polymeric substrate in 20 mm Tris and 20 mm NaCl (pH 7.4) was incubated at 30 °C for 30 min. The reaction was terminated by boiling at 100 °C for 1 min. The amount of acetic acid was determined using the K-ACETRM kit. For oligomeric substrates, Michaelis–Menten parameters were determined. The reaction components were incubated in a final volume of 100 μl at 30 °C for 2 h in the presence of 20 mm Tris (pH 7.4) and 20 mm NaCl, 6 μm enzyme, and 1–10 mm (GlcNAc)_1–6_. Then enzyme reaction was stopped by boiling at 100 °C for 1 min, and the concentration of released acetic acid was then measured by the K-ACETRM kit according to the manufacturer's protocol. Data analysis was performed with OriginPro 8.5 (OriginLab).

### BmCDA1 activation reactions

To test the activation of *Bm*CDA1 by molting fluid and CPAP3-A1, the recombinant *Bm*CDA1 protein (100 μg) was incubated with 100 μg of molting fluid and CPAP3-A1, respectively, in a total volume of 50 μl in buffer containing 20 mm Tris and 20 mm NaCl (pH 7.4) at 30 °C for multiple time intervals as followed: 0, 2, 5, 10, and 24 h. Ethylene glycol chitin and colloidal chitin were used as substrates to assess the activation of *Bm*CDA1 by molting fluid and CPAP3-A1. The molting fluid was extracted as mentioned by Qu *et al.* (50). CPAP3-A1 was recombinantly expressed and purified as previously described (44).

### ESI-MS analysis

For this experiment, 10 mm (GlcNAc)_3_ was treated with 10 μm *Bm*CDA8 in 20 mm Tris (pH 7.4) and 20 mm NaCl at 30 °C for 48 h. The samples were boiled at 100 °C for 2 min and then centrifuged at 17,000 × *g* for 10 min. Purified *Of*Hex1 (51) was added to the samples to a final concentration of 10 μm. The resulting solution was incubated at 30 °C for 48 h. NaN_3_ was added to all the samples at a final concentration of 0.03% to prevent bacteria growth. The reactions were terminated by boiling for 2 min. The samples treated with and without *Of*Hex1 were subjected to ESI-MS and recorded in both positive and negative mode using an Agilent 6224 TOF LC/MS system with a dual-nebulizer ESI source (Agilent Technology).

### In vitro GST pulldown assays

GST and GST-CPAP3-A1 were constructed and expressed in *E. coli* strain BL21 (DE3). Pulldown assay was performed using BeyoGold^TM^ GST-tag purification resin (Beyotime) according to the manufacturer's instructions. The GST proteins were incubated with 50 μl of resins in 20 mm Tris (pH 8.0), 200 mm NaCl, and 0.5% (v/v) Nonidet P-40 for 2 h at 4 °C. The resins were washed five times and then incubated with equal amounts of His-*Bm*CDA1 overnight at 4 °C. The beads were washed five times again, and the presence of His-*Bm*CDA1 was detected by Western blotting using anti-*Bm*CDA1 antibody.

### Crystallization, data collection, and structure determination of BmCDA1-CAD

Crystals of native *Bm*CDA1-CAD were grown by vapor-phase diffusion using the hanging-drop method with an equal volume (1 μl) of protein (12 mg/ml in 20 mm Tris and 20 mm NaCl, pH 7.4) and reservoir solution (0.2 m sodium malonate and 20% PEG 3350 with a final pH 7.0) at 277 K. Crystals were harvested in rayon fiber loops and bathed in a solution containing reservoir solution and 25% (v/v) glycerol as a cryoprotectant prior to flash freezing in liquid nitrogen. Diffraction data were collected using BL18U of the National Facility for Protein Science Shanghai at the Shanghai Synchrotron Radiation Facility in China. The diffraction data were processed and integrated using the HKL-3000 package (52). Data processing statistics are given in Table 1.

The structure of *Bm*CDA1-CAD could not be solved using any of the deposited CE4 structures as a search model in molecular replacement calculations. Phasing information was thus obtained using single-wavelength anomalous dispersion with selenium derivative data. The crystals of selenium-containing *Bm*CDA1-CAD protein were harvested using the same procedure under the same conditions. Diffraction data were collected using BL17U at the Shanghai Synchrotron Radiation Facility in China (53). Detection of heavy atom sites, phasing, and density modification was performed with AutoSol (54) in Phenix (55). The initial structure was built using Autobuild (56) in Phenix. COOT (57) was used to make manual corrections to the model between further cycles of refinement using Phaser (58). The selenium-containing *Bm*CDA1-CAD structure was used as the starting model for refinement of the native *Bm*CDA1-CAD data. The native *Bm*CDA1-CAD data were handled in the same way as the selenium-containing data. Final data and refinement statistics for the two structures are shown in Table 1.

### Crystallization, data collection, and structure determination of BmCDA8

The crystallization conditions of *Bm*CDA8 were screened by means of hanging-drop vapor diffusion in 96-well VDX plates at 277 K. The drop consisted of equal volumes (1 μl) of protein (10 mg/ml) and reservoir solution. The crystallization condition (0.02 m calcium chloride dihydrate, 0.1 m sodium acetate trihydrate, pH 4.6, 30% v/v (±)-2-methyl-2,4-pentanediol) was obtained from Crystal Screen 2 (Hampton Research). Crystals of *Bm*CDA8 were harvested in rayon fiber loops and flash frozen in liquid nitrogen. The diffraction data were collected on Beamline BL18U1 of the National Facility for Protein Science Shanghai at the Shanghai Synchrotron Radiation Facility in China. The data were processed and scaled using HKL3000 (52). Data analysis was performed using Phenix (55). The structure of *Bm*CDA8 was resolved by molecular replacement with Phaser (58) using native *Bm*CDA1-CAD structure as the model. The structure figures were created using the molecular visualization software PyMOL. Secondary structure topology cartoons were analyzed by Pro-origami (48). The structures were validated using the website https://validate-rcsb-1.wwpdb.org/.^3^

### MD simulation

Three independent MD simulations were performed using NAMD 2.10b2 with the force field charmm36. The systems were solvated with TIP3P water molecules, sodium ions were added to 0.15 m in water, and chloride ions were added to neutralize the system. An isothermal isobaric ensemble was employed at 310 K and 1 atm with a Langevin thermostat. Each system was equilibrated for 10 ns (the total energy was stable) with position restraints. Then 50-ns MD simulations without restraints were performed for each system. The RMSD value was calculated from protein Cα atoms superimposed on the starting structure.

## Author contributions

L. L. and Q. Y. conceptualization; L. L., Y. Zhou, Y. Q., and Q. Y. data curation; L. L., Y. Zhou, Y. Q., and Y. Zhang software; L. L. and Q. Y. formal analysis; L. L. validation; L. L. and X. G. investigation; L. L., Y. Zhou, and Y. Q. visualization; L. L., M. Q., X. G., Y. Zhang, and T. L. methodology; L. L. and Q. Y. writing-original draft; L. L. and Q. Y. project administration; M. Q., J. Y., and Q. Y. resources; M. Q., T. L., J. Y., and Q. Y. funding acquisition; Q. Y. supervision.

## Supplementary Material

Supporting Information

## Footnotes

3 The abbreviations used are: Please note that the JBC is not responsible for the long-term archiving and maintenance of this site or any other third party hosted site.

## Footnotes

2 The abbreviations used are:

CE4: carbohydrate esterase 4 family
CDA: chitin deacetylase
*An*CDA: CDA from *A. nidulans*
*Ar*CE4A: CDA from a marine *Arthrobacter* species
*Bm*CDA1: CDA 1 from *B. mori*
*Bm*CDA1-CAD: catalytic domain of CDA 1 from *B. mori*
*Bm*CDA8: CDA 8 from *B. mori*
*Cl*CDA: CDA from *C. lindemuthianum*
CPAP3: cuticular proteins analogous to peritrophin 3
*Ec*CDA: CDA from *E. cuniculi*
ESI: electrospray ionization
GlcN: glucosamine
MD: molecular dynamics
MF: molting fluid
*Of*Hex1: hexosaminidase 1 from *O. furnacalis*
RMSD: root-mean-square deviation
*Vc*CDA: CDA from *V. cholerae*
*Vp*CDA: CDA from *V. parahaemolyticus*.
