# Supplementary material for: Structural and biochemical insights into the catalytic mechanisms of two insect chitin deacetylases of the carbohydrate esterase 4 family
Source: J Biol Chem. 2019 Feb 12;294(15):5774–83. doi: 10.1074/jbc.RA119.007597 (PMC6463723; doi:10.1074/jbc.RA119.007597)
Supplement: Supporting Information [file supp_294_15_5774__index.html]

Structural and biochemical insights into the catalytic mechanisms of two insect chitin deacetylases of the carbohydrate esterase 4 family — Structure and activity of two insect chitin deacetylases — Structural and biochemical insights into the catalytic mechanisms of two insect chitin deacetylases of the carbohydrate esterase 4 family — Structure and activity of two insect chitin deacetylases — Supporting Information 

# Structural and biochemical insights into the catalytic mechanisms of two insect chitin deacetylases of the carbohydrate esterase 4 family

## Supporting Information

- Supporting Information (to be published online) - The file contains supporting results, tables and figures.
