# Supplementary material for: Structural and biochemical insights into the catalytic mechanisms of two insect chitin deacetylases of the carbohydrate esterase 4 family
Source: J Biol Chem. 2019 Feb 12;294(15):5774–83. doi: 10.1074/jbc.RA119.007597 (PMC6463723; doi:10.1074/jbc.RA119.007597)
Supplement: Supporting Information [file supp_RA119.007597_143163_1_supp_283119_pml1c4.pdf]

SUPPLEMENTAL DATA

Structure and catalysis of two chitin deacetylases from the insect *Bombyx mori* add new knowledge to the carbohydrate esterase 4 family

Lin Liu[1], Yong Zhou[1], Mingbo Qu[1], Yu Qiu[2], Xingming Guo[1], Yuebin Zhang[3], Tian Liu[1], Jun Yang[1], Qing Yang[1,4,*]

[1]State Key Laboratory of Fine Chemical Engineering, School of Life Science and Biotechnology and School of Software, Dalian University of Technology, 2 Linggong Road, Dalian 116024, China;
[2]Protein Engineering, Biologics Research, Sanofi, Bridgewater, NJ 08807, America;
[3]Laboratory of Molecular Modeling and Design, State Key Laboratory of Molecular Reaction Dynamics, Dalian Institute of Chemical Physics, The Chinese Academy of Sciences, Dalian 116024, China;
[4]Institute of Plant Protection, Chinese Academy of Agricultural Sciences, 2 West Yuanmingyuan Road, Beijing 100193, China.

Table of contents

1. Supplementary Table
2. Supplementary Figure
3. Substrate-binding Sites Predicted by MD Stimulations
4. The Construction of Nine Truncates of *Bm*CDA8

1. Supplementary Table

Table S1. Primers used in this study

For	Primer	Sequence (5'-3')
*Bm*CDA1-CAD	B1-F	GCTTACGTAGAATTCGACCCAAATAGAGCTCCG
	B1-R	ATAGTGGCCATCGTGGTGGTGGTGGTGGTGACTAGTATAGTGGCC
*Bm*CDA8	B8-F	GCTTACGTAGAATTCACTGAGCTGCCATTGGCT
	B8-R	CTGTCCAAGCGGGTGGTGGTGGTGGTGGTGACTAGTATAGTGGCC
D1	D1-innerF	GTTATGGCAGATTTTGGACCGTGGGTGCTC
	D1-innerR	GAGCACCCACGGTCCAAAATCTGCCATAAC
D2	D2-innerF	GTGCTCCCTATGATCTCCATGGTGGATGGT
	D2-innerR	ACCATCCACCATGGAGATCATAGGGAGCAC
D3	D3-innerF	TACGACTGCACCTGGCCTATGATCAGCTGG
	D3-innerR	CCAGCTGATCATAGGCCAGGTGCAGTCGTA
D4	D4-innerF	CTATGGCCTTACACTATTCCTGGACCGTGG
	D4-innerR	CCACGGTCCAGGAATAGTGTAAGGCCATAG
D5	D5-innerF	TGCACCTGGCCCACAACTAATCCAGGTCTA
	D5-innerR	TAGACCTGGATTAGTTGTGGGCCAGGTGCA
D6	D6-innerF	TGCACCTGGCCCACACTATGGCCTTACACT
	D6-innerR	AGTGTAAGGCCATAGTGTGGGCCAGGTGCA
D7	D7-innerF	GAATCTATTCAGGACCCAACTGCGTCAATT
	D7-innerR	AATTGACGCAGTTGGGTCCTGAATAGATTC
D8	D8-R	CGGGACGGGGTTGTGGTGGTGGTGGTGGTGACTAGTATAGTGGCC
D9	D9-R	AGTAAATTTGCAGTGGTGGTGGTGGTGGTGACTAGTATAGTGGCC

2. Supplementary Figure

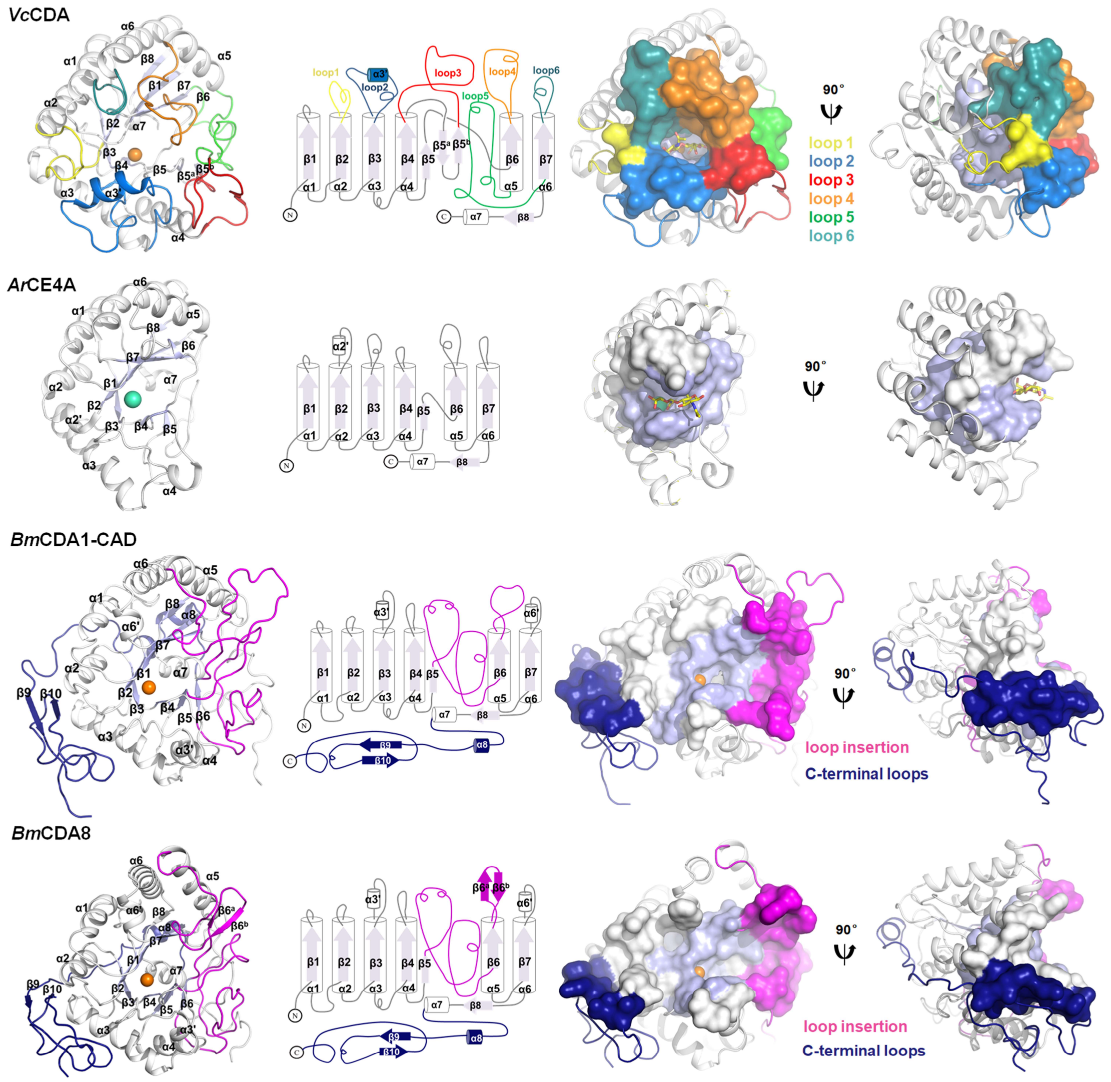

Figure S1. Structural comparison of the substrate-binding clefts among *Vc*CDA, *Ar*CE4A, *Bm*CDA1-CAD and *Bm*CDA8. The substrate-binding clefts are shown in surface representation, while the remaining regions of the enzymes are shown in cartoon representation. The active sites are shown with slate. The substrates in *Vc*CDA-(GlcNAc)$_3$ and *Ar*CE4A-(GlcNAc)$_2$ complexes are shown in stick representation with yellow carbon atoms. The zinc ion is shown as orange sphere, while the nickel ion is shown as greencyan sphere.

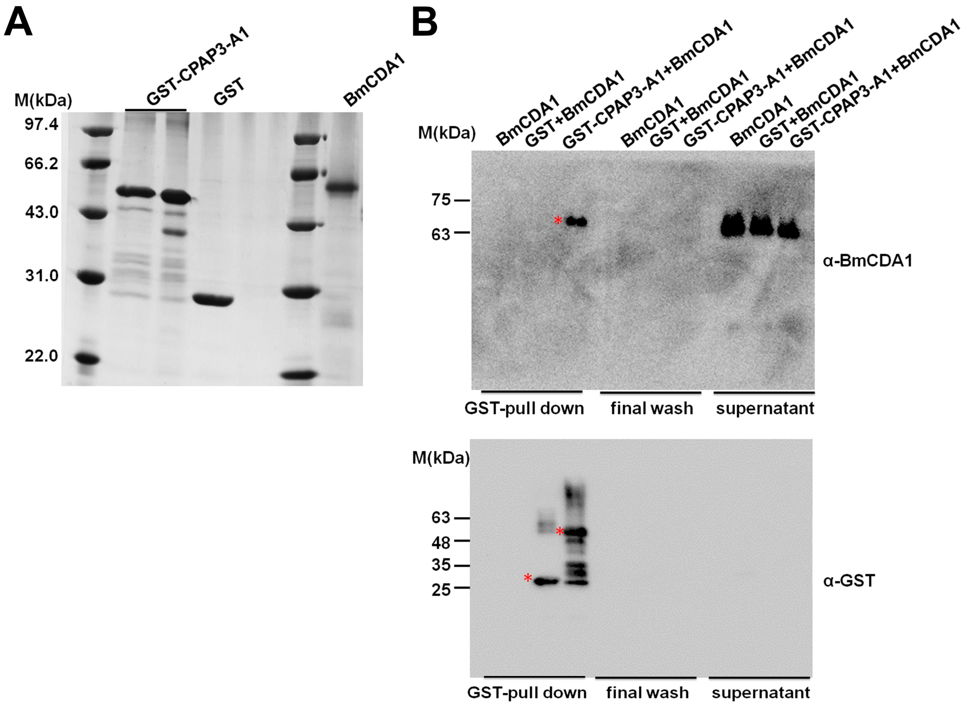

Figure S2. SDS-PAGE analysis of the GST-CPAP3-A1 fusion protein, GST and *Bm*CDA1 *(A)* and pull-down assays *(B)*. *Bm*CDA1 (~65 kDa) is specifically pulled down by GST-CPAP3-A1 fusion protein but not by GST. Markers indicate molecular weight (kDa). GST and *Bm*CDA1 alone was used as a negative control. Supernatant extracts and the final washing steps are shown as control samples. Asterisks indicate *Bm*CDA1, GST and GST-CPAP3-A1 proteins.

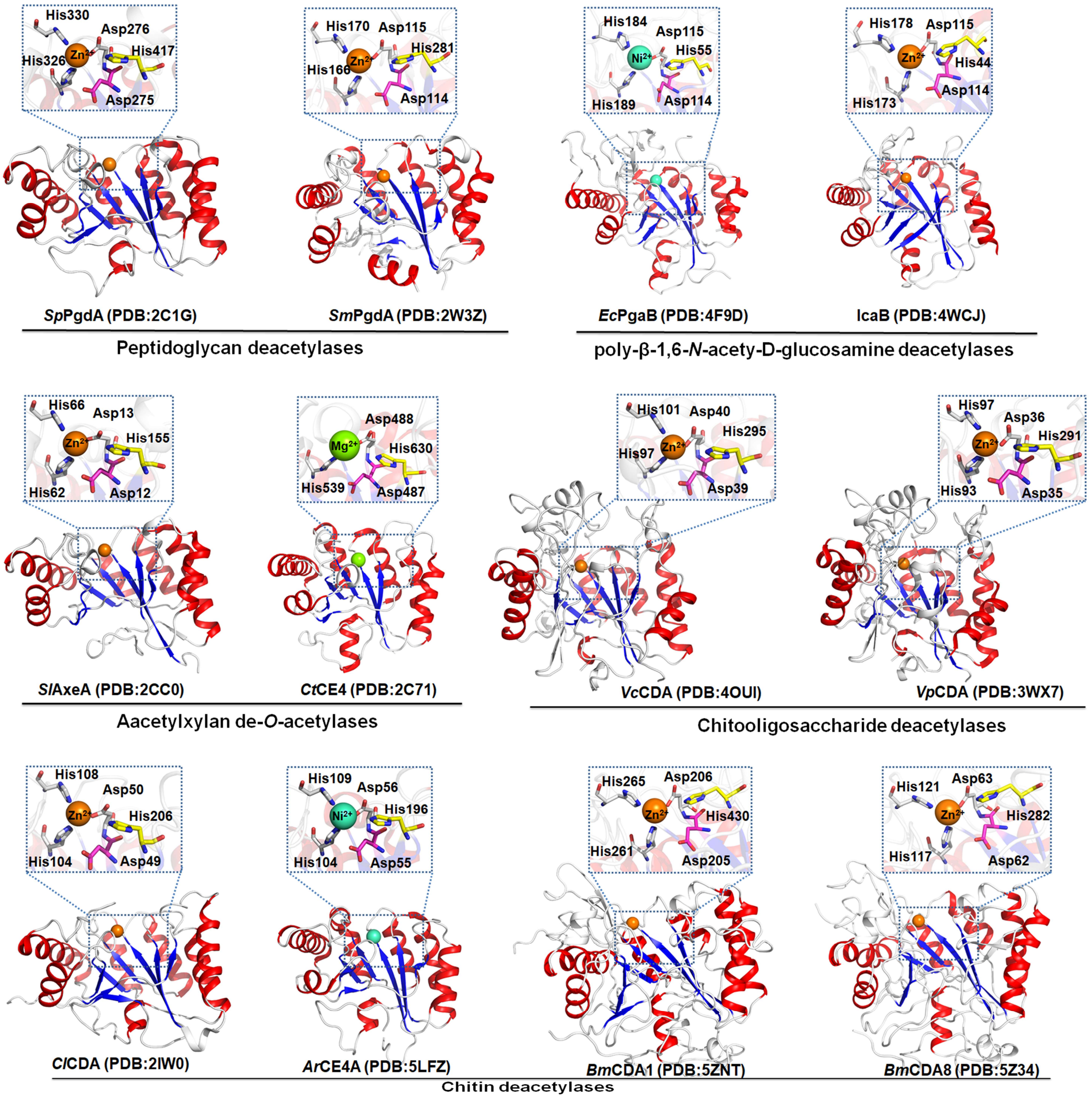

Figure S3. Structural comparison of CE4 members. These CE4 members share common (β/α)$_7$ barrel, catalytic residues and metal-binding trial. Catalytic residues are shown as sticks in a zoom to the active site.

3. Substrate-binding Sites Predicted by MD Stimulations

50 ns MD simulations were performed to visualize the predicted ligand-receptor interactions in order to investigate the detailed binding mode of $(GlcNAc)_3$ to *Bm*CDA8. The starting model of the $(GlcNAc)_3$-*Bm*CDA8 complex was built based on the homology model $(GlcNAc)_3$-*Vc*CDA (PDB entry 4OUI) and the experimental data on the deacetylation mode. The predicted interactions of the ligand are conserved during the short MD simulations. The RMSD value is approximately 1.5 Å (**Fig. S4A**).

The simulation results indicate that $(GlcNAc)_3$ binds to subsites 0, +1, +2 of *Bm*CDA8 via multiple interactions (**Fig. S4B**). In subsite 0, the acetyl moiety of the non-reducing GlcNAc residue is located in the equivalent position to that observed in the CE4-enzyme-substrate complex (15,24). In addition, the two acetate oxygen atoms of the acetyl group coordinate with a Zn^{2+} ion. In subsite +1, the acetyl moiety of the mid GlcNAc residue provides a crucial contribution to a hydrogen bond with Gln125. In subsite +2, two interactions occur between the enzyme and the reducing GlcNAc residue. The C3 hydroxyl group hydrogen bonds with the side-chain oxygen atom of Ser241, and the C5 hydroxyl group hydrogen bonds with Gln125.

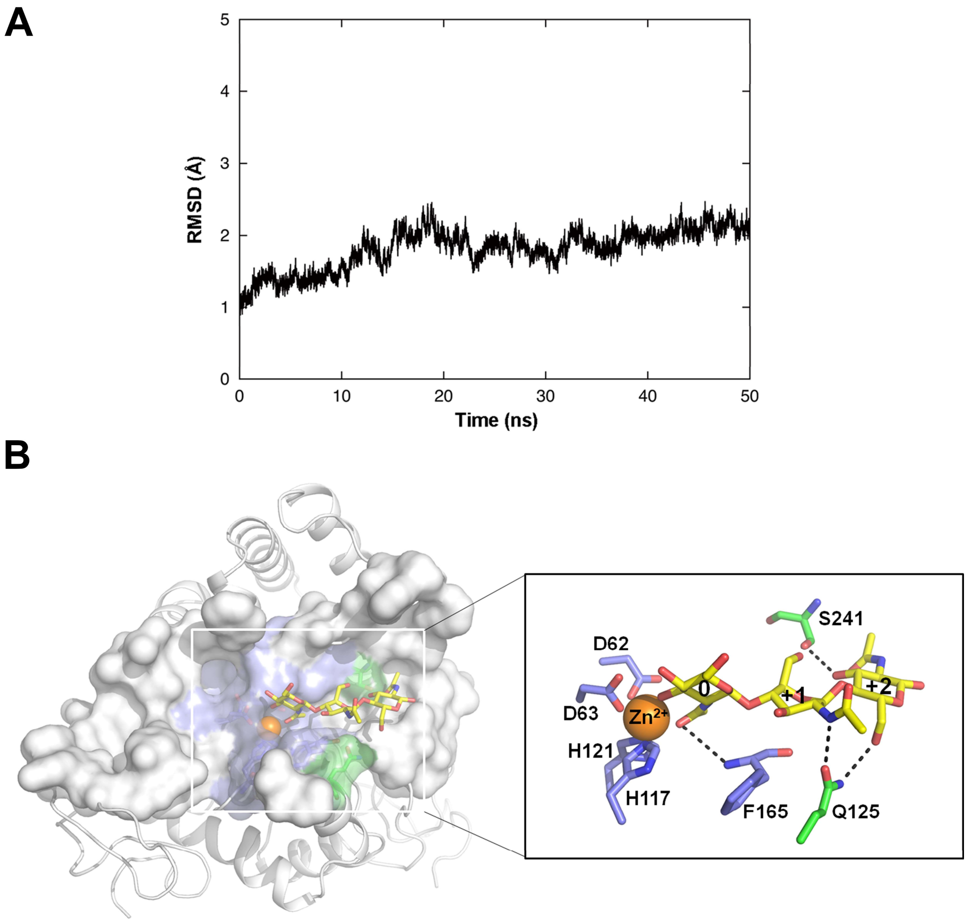

Figure S4. Substrate-binding sites predicted by MD stimulations. *(A)* RMSDs of Cα atoms of the $(GlcNAc)_3$-*Bm*CDA8 complex model during the MD simulation. *(B)* The binding mode of $(GlcNAc)_3$ to *Bm*CDA8. The substrate-binding cleft is shown in surface representation, while the remaining regions of the enzymes are shown in cartoon representation. The ligand is

shown in stick representation with yellow carbon atoms. The active site residues and other residues that interact with the ligand are shown as stick with slate and green carbon atoms, respectively. The numbers indicate the subsite to which the sugar is bound. Hydrogen bonds are shown as black dashed lines.

4. The Construction of Nine Truncates of *Bm*CDA8

To illuminate the functions of the unique loops in *Bm*CDA8, we have constructed nine truncates (D1-D9), each of which lacks partial or whole loops correspondingly. The overall strategy is summarized in **Fig. S5** and the sequences of the primers used in fragments amplification are listed in **Table S1**. The procedures of the expression plasmids construction, proteins expression and purification were the same as those described in **EXPERIMENTAL PROCEDURES**.

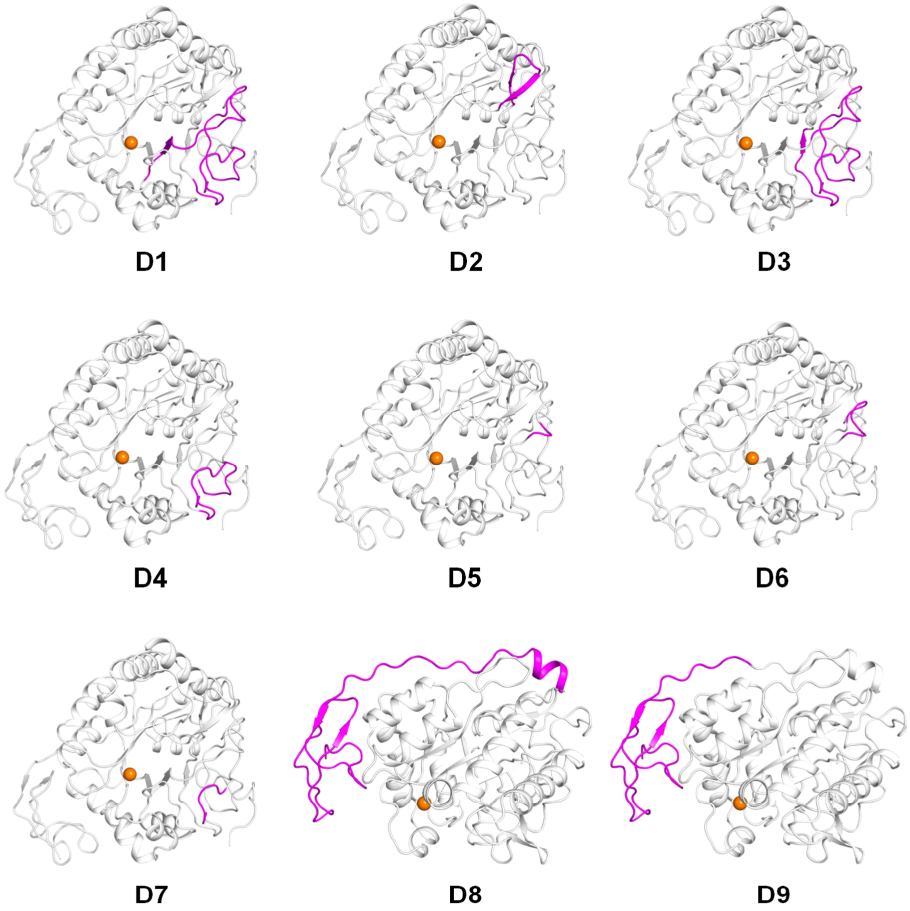

Figure S5. The overall strategy used in the construction of nine truncates of *Bm*CDA8. D1-D9 represent the nine truncates respectively. The truncated regions of *Bm*CDA8 are shown in magenta and the rest regions of *Bm*CDA8 are shown in white.
